# A smart friction control strategy enabled by CO_2_ absorption and desorption

**DOI:** 10.1038/s41598-019-49864-w

**Published:** 2019-09-13

**Authors:** Jing Hua, Marcus Björling, Mattias Grahn, Roland Larsson, Yijun Shi

**Affiliations:** 1 Division of Machine Elements, Luleå University of Technology, 97187 Luleå, Sweden; 2 Division of Chemical Engineering, Luleå University of Technology, 97187 Luleå, Sweden

**Keywords:** Chemical engineering, Mechanical engineering

## Abstract

Intelligent control of friction is an attractive but challenging topic and it has rarely been investigated for full size engineering applications. In this work, it is instigated if it would be possible to adjust friction by controlling viscosity in a lubricated contact. By exploiting the ability to adjust the viscosity of the switchable ionic liquids, 1,8-Diazabicyclo (5.4.0) undec-7-ene (DBU)/ glycerol mixture via the addition of CO_2_, the friction could be controlled in the elastohydrodynamic lubrication (EHL) regime. The friction decreased with increasing the amount of CO_2_ to the lubricant and increased after partial releasing CO_2_. As CO_2_ was absorbed by the liquid, the viscosity of the liquid increased which resulted in that the film thickness increased. At the same time the pressure-viscosity coefficient decreased with the addition of CO_2_. When CO_2_ was released again the friction increased and it was thus possible to control friction by adding or removing CO_2_.

## Introduction

Friction is the force resisting the relative motion of solid surfaces or fluid layers sliding against each other. Often, we try to minimize friction but there are also many situations where high friction is desirable. While in some cases something in between, *i.e*. optimum friction is desirable. Nowadays both ultrahigh friction^1^ and ultralow friction^2–5^ have been studied extensively. Ultrahigh interlayer friction was observed when Niguès *et al*.^1^ investigated the friction performance of multiwalled boron nitride nanotubes (BNNTs), caused by the structural reorganization of the BNNTs layer. On the other side, not only at nano- or microscale^2–4^, but also at macroscale^5^, ultralow friction can be attained. It was shown that highly oriented pyrolytic graphite, diamondlike carbon films and molybdenum disulfide can lead to ultralow friction under special lubrication conditions. Compared with achieving extremely high or low friction, the intelligent control of tribological interactions is also attractive^6–9^. For example, friction control by light can be used to manipulate the gripping friction of a robotic finger^9^, in which the friction drops by a factor of four to five when the light is switched on.

Some other methods have also been employed to control friction^10,11^. Meng *et al*.^10^ found that the friction coefficient of the brass/silicon dioxide couple sliding material pair was controllable between 0.06 to 0.21 by applying a certain range electric voltage on the material pair measured by a self-made friction tester under a load of 9.8 N (Hertzian contact pressure ≈ 610 MPa). The friction of poly methylacrylic acid sodium (PMAA) brushes can be controlled via pH value under a constant load of 0.5 N (Hertzian contact pressure ≈ 0.23 MPa)^11^. PMAA showed ultralow friction coefficient (μ ∼ 0.006) under both neutral (pH ≈ 7) and basic (pH ≈ 12) solutions whereas at low pH value (pH ≈ 2), the negative carboxylate groups were rapidly protonated and the PMAA brush fully collapsed and dehydrated, which led to ultrahigh friction (μ > 1). However, one of the limitations of these studies is that the loads in these experiments are quite low. Instead, running experiments with full size from the real application has the advantage of giving realistic results in terms of power losses depending on lubricant type, load, speed and operating temperature^12^.

The atmosphere at which experiments are conducted may affect the friction in dry lubrication^13,14^, thus it has been demonstrated that it is possible to control friction via altering the atmosphere. Mishina *et al*.^13^ studied the effect of the composition of the gas atmosphere on friction and adhesive wear of six different pure metals and Zhang *et al*.^14^ found that the diamond-like carbon (DLC) film shows significantly different friction characteristics under different atmosphere. The influence of the atmosphere on the friction behavior of fluid lubrication has been studied as well. Lee *et al*.^15^ revealed that friction coefficient and the amount of wear of sliding surfaces in the compressor became larger due to the increased pressure of CO_2_, when polyol ester was used as lubricant. Their studies, however, did not address the possibility of friction control by atmosphere environment.

It is reported that the viscosity of the switchable ionic liquids can be increased by up to an order of magnitude after absorbing CO_2_^16^.Viscosity is the most essential feature of lubricants, as it enables them, given the right conditions, to separate two solid bodies in relative motion^17^. Switchable ionic liquids /CO_2_ binding organic liquids(CO_2_BOLs) are the mixtures of an alcohol and an amidine or guanidine, based on Jessop’s switchable solvent^18–20^, which can capture and release CO_2_ efficiently. The mechanism of capturing CO_2_ is that an amidinium or guanidinium alkylcarbonate salt is formed after absorbing CO_2_. By heating or bubbling another gas, the CO_2_ will be reversibly released and the low viscosity liquid is obtained again. Inspired by the controllable change in viscosity of switchable ionic liquid under CO_2_ atmosphere, it seems that CO_2_ has the potential to be used to control tribological performance. Pohrer *et al*.^21^ found a significant change in viscosity after absorbing CO_2_ but the change of viscosity could not affect the friction property. The possible explanation is that the friction test in their work was operating in boundary lubrication where viscosity of lubricants has almost no significant impact on friction. Elastohydrodynamic lubrication (EHL) is a more appropriate lubricating regime to evaluate the influence of viscosity on friction, since the viscosity is considered to be one of the best single parameters for assessing the performance of fluid film lubrication.

In this article, switchable ionic liquids, able to capture CO_2_ in a controlled manner, were used as lubricants. The idea was to investigate if it would be possible to adjust friction by controlling viscosity in a lubricated contact between a ball and disc operating under elastohydrodynamic lubrication (EHL) conditions. The ability to adjust the viscosity of switchable ionic liquids was exploited by studying mixtures of glycerol and 1,8-Diazabicyclo (5.4.0) undec-7-ene (DBU) while CO_2_ was added. It was shown that the viscosity of the mixture increased when CO_2_ was absorbed. The higher viscosity mixtures were shown to increase film thickness and reduce friction under EHL. The friction reduction is partly attributed to the decrease of the fluids pressure-viscosity behavior with increasing CO_2_-content. The effect was reversed when CO_2_ was released. Altogether it is shown that the absorption and release of CO_2_ in a switchable ionic liquid can be used for online control of friction and fluid film under EHL.

## Results

### CO_2_ absorption and desorption

The absorption loading of CO_2_ in the studied mixtures was calculated using the following equations:

1 $${n}_{C{O}_{2}}=\frac{{P}_{0}({V}_{A}-{V}_{L})}{{Z}_{1}RT}-\frac{{P}_{t}({V}_{A}-{V}_{L})}{{Z}_{2}RT}$$

2 $$z=1+\frac{BP}{RT}$$

3 $$x=\frac{{n}_{C{O}_{2}}}{{n}_{DBU}}$$

where *P*_0_ and *P*_*t*_ are the initial and instantaneous pressures, respectively. Comparing with the operating pressure, the saturated vapor pressure of the mixtures can be neglected. *V*_*A*_ and *V*_*L*_ represent the volumes of the absorption vessel and mixtures. *Z*_1_ and *Z*_2_ are the compressibility factors corresponding to the initial and instantaneous pressure, respectively. The generalized second virial coefficient correlation was used to get an approximation of compressibility factor *Z*. In equation (2), *B* is the correlation to critical properties of CO_2_. ${n}_{C{O}_{2}}$ is the number of moles of CO_2_ absorbed by the switchable ionic liquids. *n*_*DBU*_ is the molar amount of DBU, equal to the theoretical amount of absorbed CO_2_ and, finally, *x* is the absorption loading.

**Figure 1:**
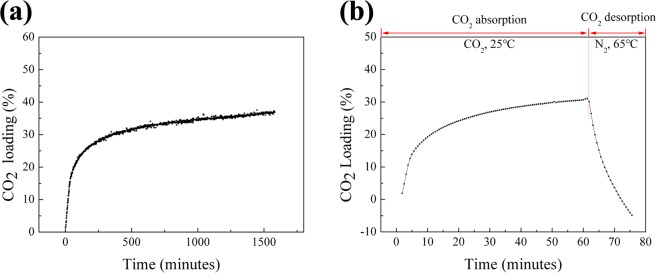
(**a**) CO_2_ absorption kinetics of mixture of DBU and glycerol in 3:1 molar ratio at 25 °C; (**b**) CO_2_ absorption and desorption on the mixture of DBU and glycerol in 3:1 molar ratio by TGA (absorption at 25 °C for 60 min; desorption by N_2_ at 65 °C for 15 min).

As shown in Fig. 1(a), the CO_2_ loading of DBU/glycerol mixture with 3:1 molar ratio increases as a function of time. The CO_2_ loading increased rapidly when the loading was below 20%, then the absorption rate decreased gradually. This is mainly because the viscosity of mixtures at high loading is too large to be stirred magnetically, resulting in a mass-transfer limitation^22^. This is the main reason we choose the low CO_2_ loading for friction study in the coming part. Figure 1(b) shows the TGA curve for the mixture of DBU and glycerol in 3:1 molar ratio as a function of time. After 1 hour’s absorption, the maximum CO_2_ loading achieved 32% of theoretical loading and change of absorption rate appeared to be similar with that present in Fig. 1(a). The decomposition temperature of DBU/glycerol/CO_2_ is 60 °C^23^, and the desorption only took approximately 15 minutes at 65 °C. The weight loss was even larger than the weight of CO_2_ absorbed after 10 minutes’ desorption, which indicated that part of the DBU was evaporated during the TGA measurement.

### Viscosity

**Figure 2:**
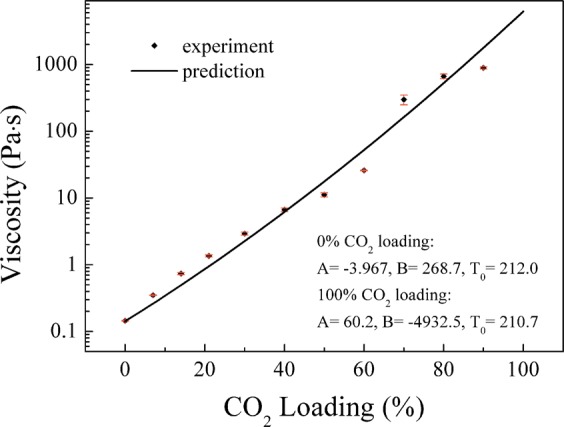
Viscosity as function of CO_2_ loading at 25 °C.

The most accurate three-parameter equation for describing the viscosity, named Vogel-Fucher-Tammann-Hesse (VFTH), was used to describe the viscosity of each component^24^, see equation 4. Then a simplified two-component model was used to predict the viscosity via Eq. (5). Figure 2 shows how the viscosity increases as a function of CO_2_ loading. Like all studied switchable ionic liquids, an increase in viscosity can be observed after absorbing CO_2_ since mass-transfer limitations are easily introduced^16^. According to Ikenna’s study, the highest viscosity of DBU/glycerol mixture at 25 °C is 160 *Pa* · *s*^23^, which is much lower than the results presented here. This is mainly because the high viscosity results in an absorption rate so low that higher CO_2_ loading (>90%) could not be achieved in short time by using magnetic stirrer. There is one thing needs to be pointed out here that when CO_2_ loading is above 30%, DBU/glycerol/CO_2_ mixtures become too viscous for the pump to cycle them for the friction test.

4 $$\log \,\mu =A+\frac{B}{T-{T}_{0}}$$

5 $$\log \,\mu ={\sum }_{i=1}^{n}{A}_{i}{X}_{i}+\frac{{\sum }_{i=1}^{n}{B}_{i}{X}_{i}}{T-{\sum }_{i=1}^{n}{T}_{{0}_{i}}{X}_{i}}$$

where *X*_*i*_ is the relative concentration of each component.

**Figure 3:**
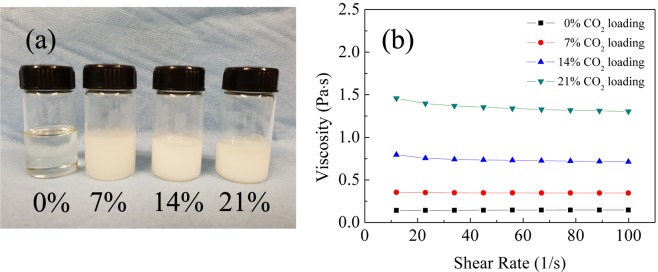
(**a**) Photos of DBU/glycerol mixtures with different loading of CO_2_, (**b**) Viscosity at different shear rates after CO_2_ absorption at different levels in DBU/glycerol mixtures.

Considering the absorption/desorption and viscosity properties of DBU/glycerol/CO_2_ mixtures, four mixtures with lower CO_2_ loadings (0%, 7%, 14% and 21%) were prepared to investigate the tribological properties. Photos of these samples are shown in Fig. 3(a). It is obvious that absorption of CO_2_ has a strong effect on viscosity. The viscosity of the liquid increases with increasing CO_2_ loading (Fig. 3(b)), from 0.15 *Pa* · *s* to 1.3 *Pa* · *s*, which stays in a good viscosity range for the EHL friction study. The viscosity is relatively insensitive to shear rate in the measured range as illustrated in Fig. 3(b), which will ensure a good friction measurement.

### Friction test

**Figure 4:**
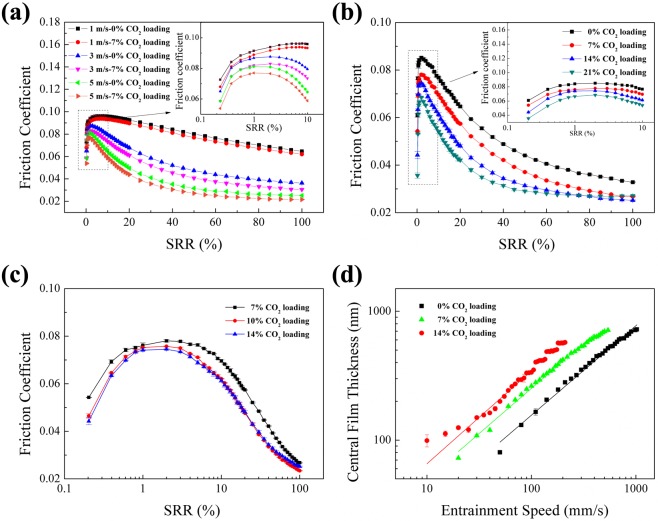
(**a**) Friction coefficients as function of SRR and entraining velocity at a contact pressure of 1.35 GPa; (**b**) Friction coefficients at an entrainment speed of 3 m/s and a contact pressure of 1.35 GPa for liquids with different CO_2_ loading as function of SRR; (**c**) Friction coefficients at an entrainment speed of 3 m/s and a contact pressure of 1.35 GPa for liquids with different CO_2_ loading at low SRR; (**d**) Central film thickness as function of entrainment speed in EHL contact for DBU/glycerol/CO_2_.

The first EHL study was a qualitative analysis with the aim of capturing general trends of the friction coefficient with and without CO_2_. Different slide-to-roll ratios (SRR) and entrainment velocities were used and the result is presented in Fig. 4(a). Friction coefficients versus SRR and entraining velocities were measured with fluids without CO_2_ and with approximately 7% CO_2_ loading. The results clearly show that introducing CO_2_ to the mixture reduced friction at all investigated entrainment speeds.

EHL friction is greatly influenced by the pressure-viscosity behavior of the lubricant. When CO_2_ is added, it is likely that the pressure-viscosity coefficient is reduced and thus also friction. The film thickness increase itself may also alter the friction since the average Couette shear rate is reduced when the film becomes thicker.

To better understand the role of CO_2_ in controlling friction, the prepared DBU/glycerol mixtures with different CO_2_ loading (0%, 7%, 14% and 21%) were used for studying the relationship between the friction coefficient and CO_2_ loading. The slide-to-roll ratio was varied whereas the contact pressure and entrainment speed were kept constant at 1.35 GPa and 3 m/s, respectively. The recorded friction coefficients as a function of CO_2_ concentration in the lubricants are shown in Fig. 4(b). At low SRR it is again shown that the friction coefficient is reduced with increasing CO_2_ concentration. The trend was very similar at high SRR’s with decreasing friction with increasing concentration of CO_2_, except for the specimen of 21% CO_2_ loading where the friction remained more or less constant at SRR higher than 75%. Here, the friction becomes larger than the mixtures with 7% and 14% CO_2_ loading. This may be explained by the contradicting effects of CO_2_ and SRR.

The friction behavior of specimens after CO_2_ desorption was investigated as well. In order to enhance the desorption of CO_2_, heating, stirring and bubbling with N_2_ gas were applied. The regeneration temperature was set to 65 °C, same as the desorption temperature in TGA. After half an hour’s desorption, the CO_2_ loading decreased from 14% to 10%, and the friction behavior before and after desorption are shown in Fig. 4(c). For the mixture with partial desorption CO_2_, the expected increase of friction occurred at low SRR.

## Discussion

When absorbing CO_2_, the friction of the specimen of 21% CO_2_ loading becomes larger than the mixtures with 7% and 14% CO_2_ loading at high SRR. It may be explained by the contradicting effects of CO_2_ and SRR. The viscosity becomes higher as CO_2_ loading increases but at higher SRR the high shearing may reduce the viscosity at the inlet to the EHL contact and thus also reduce the film thickness, with an increase in friction as a result. Yet, another possible explanation is that the switchable ionic liquids decompose at high shear rate. The decomposition temperature of DBU/glycerol/CO_2_ is 60 °C^23^, which would indicate that the energy required in this decomposition reaction to break down the bonds present in the substance is not very high. It is possible that this temperature can be reached locally in the high-pressure contact^25^.

In order to understand more about the lubricating mechanism of DBU/glycerol/CO_2_ mixture, the central film thickness of the lubricants as a function of the entrainment speed was investigated. This requires the refractive index of the lubricants to be known. Consequently, the refractive index of the lubricants was measured and the values obtained are presented in Supplementary Table 1. As shown, the effect of CO_2_ is very small and can be neglected.

The central film thickness for the different lubricants is shown in Fig. 4(d). At a CO_2_ loading below 14%, the central thickness increases linearly with increasing entrainment speed when plotted on a logarithmic scale. For the sample with 14% CO_2_ loading, the central thickness shows a linear relationship when the entrainment speed is lower than 100 mm/s. As it can be seen in Fig. 3(a), the opacity of the fluid is increased with higher CO_2_ loading. This creates a problem with the interferometry measurements and the mixture with 21% CO_2_ loading could not be measured.

The experimental data were fitted using the following power function which is derived from the Hamrock-Dowson equation^26^:

6 $${H}_{C}=a\times {U}_{e}^{b}$$

where *H*_*C*_ is the central film thickness (nm), *U*_*e*_ is the entrainment speed (mm/s), *a* and *b* are constants. For 0% CO_2_ loading, 7% CO_2_ loading and 14% CO_2_ loading, the film thickness increased with the entraining speed at a rate of ${U}_{e}^{0.69}$, ${U}_{e}^{0.70}$ and ${U}_{e}^{0.72}$ respectively. This indicates that the lubrication film formation of DBU/glycerol mixture is slightly more sensitive to the entrainment speed at higher CO_2_ loadings.

As mentioned, the pressure-viscosity relationship has a large impact on the viscosity inside the EHL contact, and thus also the EHL full film friction. Let us, for simplicity, use the concept of a pressure-viscosity coefficient, see for example the work of Hamrock and Dowson^26^. According to their film thickness equation, the pressure-viscosity coefficient is one of the most important factors that affect the film thickness in the EHL regime. Based on Van Leeuwen’s work^27^, the Hamrock–Dowson equation can be used to calculate the pressure–viscosity coefficient, α, from film thickness data and this equation is considered to be superior to other equations used. Therefore, the Hamrock–Dowson equation^26^ was employed here to calculate the α-value of the different DBU/glycerol/CO_2_ solutions. The Hamrock–Dowson central film thickness equation is defined as follows:

7 $${H}_{C}=1.345{R}_{x}{U}^{0.67}{G}^{0.53}{w}^{-0.0067}{C}_{o}$$

where

8 $$U=\frac{{\eta }_{o}{U}_{e}}{{R}_{x}E^{\prime} }$$

9 $$W=\frac{2\cdot {F}_{N}}{{R}_{x}^{2}\cdot E^{\prime} }$$

10 $$G=2\alpha E^{\prime} $$

11 $${C}_{o}=1-0.61{{\rm{e}}}^{-0.752{({R}_{x}/{R}_{y})}^{0.64}}$$

12 $${U}_{e}=\frac{{U}_{1}+{U}_{2}}{2}$$

where *R*_*x*_ is the radius of curvature of the ball in -x-direction, *R*_*y*_ is the radius of curvature of the ball in -y-direction, *U* is the dimensionless speed parameter, *U*_1_ is the ball speed (mm/s), *U*_2_ is the disc speed (mm/s), *G* is the materials parameter, *W* is the load parameter, *C*_*o*_ is the ellipticity influence, *η*_0_ is the viscosity at atmospheric pressure (Pas), *E*′ is the equivalent Young modulus (Pa), *F*_*N*_ is the normal force (N) and *α* is the pressure–viscosity coefficient (Pa^−1^). Since all parameters, except the pressure-viscosity, are known it is possible to compute α. One must, however, understand that effects of inlet shear thinning and heating are not taken into account and this implies that the absolute value of α may be affected by relatively large errors. Still it is possible to compare the computed values obtained in the same setup.

The calculated pressure–viscosity coefficients for the lubricants are listed in Supplementary Table 2. The pressure-viscosity coefficient decreases with increasing CO_2_ loading, which may be one of the reasons for the observed trend with lower friction coefficient with increasing CO_2_ loadings. A lower pressure-viscosity coefficient will lead to lower local viscosity in the high-pressure zone of the contact and therefore lower friction. Low pressure-viscosity has been given as an explanation for low friction in other fluids, for example glycerol^28,29^.

In conclusion, a novel lubricant with controllable friction properties has been investigated. The friction control is obtained by adding CO_2_ to the lubricant. The modulation of friction is based on the switch of viscosity depending on CO_2_ loading. It was demonstrated that the viscosity can be altered and that the lubricant does not suffer the problem of high mass transfer resistance at room temperature when the CO_2_ loading is less than 21%. The EHL friction in ball-on-disc test switches to a lower value when CO_2_ is absorbed, and it turns higher when CO_2_ is released. This study has also shown that in pure rolling condition, with higher CO_2_ loading, the film thickness will be increased and the pressure-viscosity coefficient will be decreased, which are believed to be the main reasons for the observed reduction of friction.

## Methods

### Sample preparation

The physical properties of switchable ionic liquids can be manipulated by changing the alcohol used, e.g. by using glycerol, 1-hexanol, 6-amino-1-hexanol, L-prolinol and so on. Glycerol can be looked upon as a green lubricant, which can generate a very low friction coefficient^28,30–32^. In this work, glycerol (Aldrich, 99 + %) and 1,8-Diazabicyclo (5.4.0) undec-7-ene (DBU) (Aldrich, 98%) were selected as the alcohol and the base, respectively.

The DBU glycerol carbonate was prepared via bubbling of CO_2_ through a 3:1 molar ratio solution of DBU and glycerol. DBU (127.38 g) and glycerol (25.42 g) were used when preparing a 3:1 molar ratio of the mixture. Thereafter, a narrow tube was inserted and CO_2_ (AGA, 99.7+%, H_2_O < 100 ppm) was bubbled through the liquid. The reaction was exothermic and the mixture was stirred mechanically throughout the bubbling cycle. The CO_2_ loading was calculated by the weight increment. The accuracy of the analytical balance used was 0.01 g. The water content was checked with a Karl Fisher titration device to be less than 0.5 wt% before and after each test.

### CO_2_ absorption and desorption

The experimental apparatus for measuring the gas absorption loading has been described previously^33^. It contains a gas reservoir, an absorption vessel, a magnetic stirrer and two pressure transducers. The water bath was heated to the set temperature (25 °C) and maintained for 1 hour. An accurate amount (3.3 ml) of a 3:1 molar ratio solution of DBU and glycerol was added into the absorption vessel. The dissolved gas was removed by a vacuum pump. Thereafter an accurate amount of pure CO_2_ gas was pumped in to absorption vessel, and the pressure decrease was recorded.

In order to evaluate the performance of DBU/glycerol mixtures on CO_2_ absorption and desorption, a thermogravimetric analyzer (TGA, Perkin Elmer 8000) was used. The initial activation of the sorbent was carried out by heating 5.127 mg of sample loaded in the platinum pan of the thermal analyzer, to 25 °C in pure nitrogen (99.99% purity) at a flow rate of 20 ml/min. Pure CO_2_ (99.99% purity) was then introduced at the same flow rate and the temperature was kept at 25 °C. The CO_2_ absorption rate of the sample was calculated from the mass gain after holding it at 25 °C in CO_2_ for 60 minutes. Subsequently, the desorption of CO_2_ was carried out by increasing the temperature to 65 °C for 15 minutes in an atmosphere of pure N_2_ at a flow rate of 20 ml/min.

### Viscosity

The viscosities of the DBU/glycerol/CO_2_ mixtures were measured at 25 °C using a CVO Bohlin Rheometer. The low viscosity samples were measured with the bob/cup geometry, *i.e*. cylinder in cylinder geometry with a clearance of 1.25 mm.

The shear rate was increased logarithmically from 0 to 100 s^−1^. The high viscosity samples with the cone/plate geometry, cone-angle = 1°, cone radius *R* = 10 mm, at a constant shear rate of 10 s^−1^.

### Friction tests

The EHL friction behavior of the DBU/glycerol/CO_2_ mixtures was investigated in a WAM (Wedeven Associates machine) ball-on-disc test apparatus, model 11A, at room temperature (ca 25 °C), at a load of 100 N (1.35 GPa maximum Hertzian pressure), with an entrainment speed between 1–5 m/s and a slide-to-roll ratio (SRR) between 0–100%. Before each test, the device and specimens were thoroughly cleaned with acetone and ethanol. Thereafter, the specimens, the ball and the disc were assembled. Then the relative position of the ball and disc was corrected to pure rolling and the desired slide-roll ratio was set. The disc was constantly lubricated using a recirculation pumping system attached to the test rig. A full description of this equipment has been reported previously^34^.

All specimens used in the tests, both balls and disc were made from AISI 52100 bearing steel. The balls were taken directly from the factory and the disc was processed in the same way as bearing raceway material. The balls were grade 20 with a surface roughness (Ra) of 30 nm, an outer diameter of 20.637 mm, and a hardness of about 60 HRC. The disc had a surface roughness (Ra) of 90 nm, 101.6 mm as outer diameter, a circumferential grind and were through hardened to about 60 HRC.

### Film thickness investigation

The WAM-11 was used to investigate the formation of a lubricating film for the DBU/glycerol/CO_2_ mixtures. A super-polished steel ball with a roughness of 10 nm (Ra) was brought into contact and loaded against a glass disc coated with a chromium semi-reflecting layer with a roughness of 1 nm (Ra). The chromium layer acts as a beam splitter to enable interferometric measurements of film thickness. The disc and the ball were driven by separate motors. During the tests, the steel ball was partially submerged in the lubricant. The lubricant was entrained into the contact by the ball and formed a lubricating film. A full description of this process has been reported previously^35^.

A microscope and a CCD camera were used to capture the chromatic interference pattern which occurs when the contact was illuminated by white light. The thickness of the lubricant film formed in the contact area was calculated by the interference color evaluation technique^36^. The setup made it possible to measure the film thicknesses between 80 nm and 800 nm. The elastic modulus and Poisson’s ratio were 64 GPa and 0.2 respectively for the glass disc, and 210 GPa and 0.3 respectively for the AISI 52100 steel ball. The outer diameter of the steel ball was 20.625 mm and that of the glass disc was 114.3 mm. Since the glass supports a maximum Hertz pressure of approximately 0.6 GPa, a load of 25 N was applied to generate a maximum Hertzian contact pressure of 0.5 GPa and a mean contact pressure of 0.33 GPa in this study. The disc and the ball were run in the pure rolling mode, and the entrainment speed was in the range 0.02–1.1 m/s. All tests were carried out at room temperature (ca 25 °C).

Before each test, the refractive index (n) of the lubricant was determined with a Zeiss Abbe Refractometer.

## Supplementary information

Supplementary Information
