# Supplementary material for: A smart friction control strategy enabled by CO2 absorption and desorption
Source: Sci Rep. 2019 Sep 13;9:13262. doi: 10.1038/s41598-019-49864-w (PMC6744455; doi:10.1038/s41598-019-49864-w)
Supplement: Supplementary file 1 — Supplementary Information [file 41598_2019_49864_MOESM1_ESM.docx]

**A smart friction control strategy enabled by CO_2_ absorption and desorption**

# Jing Hua^1^, Marcus Björling^1^, Mattias Grahn^2^, [Roland Larsson](https://www.sciencedirect.com/science/article/pii/S026635381732746X?via%3Dihub#!)^1^, Yijun Shi^1*^

*^1^ Division of Machine Elements,* *Luleå University of Technology, 97187, Luleå, Sweden*

*^2^ Division of Chemical Engineering,* *Luleå University of Technology, 97187, Luleå, Sweden*

*Correspondence to yijun.shi@ltu.se

**Supplementary Information**

**Supplementary** **Table. 1.** Refractive index of the lubricants at different CO_2_ loading.

| lubricant | 0% CO_2_ loading | 7% CO_2_ loading | 14% CO_2_ loading | 21% CO_2_ loading |
| --- | --- | --- | --- | --- |
| Refractive index | 1.517 | 1.518 | 1.520 | 1.520 |

**Supplementary** **Table. 2.** Calculated pressure–viscosity coefficients for DBU/glycerol/CO_2_ mixtures with different CO_2_ loading.

| lubricant | 0% CO_2_ loading | 7% CO_2_ loading | 14% CO_2_ loading |
| --- | --- | --- | --- |
| *α* (×10^−9^ Pa^−1^) | 23.31 | 19.07 | 17.59 |
